# Folate metabolism: a re-emerging therapeutic target in haematological cancers

**DOI:** 10.1038/s41375-021-01189-2

**Published:** 2021-03-11

**Authors:** Martha M. Zarou, Alexei Vazquez, G. Vignir Helgason

**Affiliations:** 1 Wolfson Wohl Cancer Research Centre, Institute of Cancer Sciences, University of Glasgow, Glasgow, UK; 2 Cancer Research UK Beatson Institute, Glasgow, UK

**Keywords:** Chemotherapy, Cancer metabolism

## Abstract

Folate-mediated one carbon (1C) metabolism supports a series of processes that are essential for the cell. Through a number of interlinked reactions happening in the cytosol and mitochondria of the cell, folate metabolism contributes to de novo purine and thymidylate synthesis, to the methionine cycle and redox defence. Targeting the folate metabolism gave rise to modern chemotherapy, through the introduction of antifolates to treat paediatric leukaemia. Since then, antifolates, such as methotrexate and pralatrexate have been used to treat a series of blood cancers in clinic. However, traditional antifolates have many deleterious side effects in normal proliferating tissue, highlighting the urgent need for novel strategies to more selectively target 1C metabolism. Notably, mitochondrial 1C enzymes have been shown to be significantly upregulated in various cancers, making them attractive targets for the development of new chemotherapeutic agents. In this article, we present a detailed overview of folate-mediated 1C metabolism, its importance on cellular level and discuss how targeting folate metabolism has been exploited in blood cancers. Additionally, we explore possible therapeutic strategies that could overcome the limitations of traditional antifolates.

## Introduction

Cancer cells undergo metabolic changes to harness nutrients and support their rapid growth and proliferation. Such metabolic reprogramming has been extensively studied. The first evidence for changes in cancer metabolism were observed by Otto Warburg during the early 20th century. Warburg described an increase in aerobic glycolysis, ‘Warburg effect’, where cancer cells took up and fermented glucose to lactate in the presence of oxygen. Metabolic alterations have been exploited by researchers ever since, in order to understand tumour growth and metastatic dissemination [1, 2].

Folate metabolism, considered the core of a broader set of transformations known as one-carbon (1C) metabolism, allows the transfer of 1C units for biosynthetic purposes such as de novo synthesis of adenosine, guanosine and thymidylate and for methylation reactions that support the methionine cycle. As animals cannot synthesise folate, insufficient dietary intake can cause neural tube defects in developing foetuses and anaemia [3–5].

**Fig. 1:**
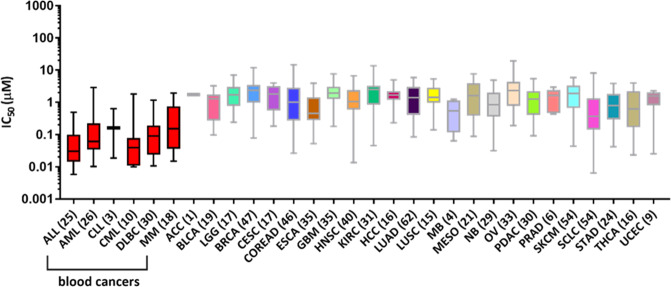
Haematological cancer cell lines show high sensitivity to methotrexate. IC50 values for methotrexate across several cell lines as obtained by the publicly available database Genomics of Drug Sensitivity in Cancer (https://www.cancerrxgene.org/). Number in parenthesis represents number of cell lines analysed per each cancer type. ALL, acute lymphoblastic leukaemia; AML, acute myeloid leukaemia; CLL, chronic lymphoblastic leukaemia; CML, chronic myeloid leukaemia; DLBC, diffuse large B-cell lymphoma; MM, multiple myeloma; ACC, adrenocortical carcinoma; BLCA, bladder urothelial carcinoma; LGG, brain lower grade glioma; BRCA, breast invasive carcinoma; CESC, cervical squamous cell carcinoma and endocervical adenocarcinoma; COREAD, colon and rectum adenocarcinoma; ESCA, oesophageal carcinoma; GBM, glioblastoma multiforme; HNSC, head and neck squamous cell carcinoma; KIRC, kidney real clear cell carcinoma; HCC, hepatocellular carcinoma; LUAD, lung adenocarcinoma; LUSC, lung squamous cell carcinoma; MB, medulloblastoma; MESO, mesothelioma; NB, neuroblastoma; OV, ovarian serous cystadenocarcinoma; PDAC, pancreatic adenocarcinoma; PRAD, prostate adenocarcinoma; SKCM, skin cutaneous melanoma; SCLC, small cell lung carcinoma; STAD, stomach adenocarcinoma; THCA, thyroid carcinoma; UCEC, uterine corpus endometrial carcinoma.

With regards to the association of the folate metabolism to blood cancers, the idea that antagonists of folates could reduce the proliferation of malignant cells in paediatric leukaemia gave rise to modern cancer therapy and a class of drugs known as antifolates [6]. Since then, methotrexate (MTX), likely the most well-known antifolate, has been used to treat a variety of neoplastic and inflammatory diseases. Analysis of publicly available data on the sensitivity of almost 700 cancer cell lines to MTX demonstrates that haematological cancer cell lines show high sensitivity to this antifolate, with acute lymphoblastic leukaemia (ALL) and acute myeloid leukaemia (AML) cell lines displaying the highest sensitivity (Fig. 1) [7]. These data suggest that targeting 1C metabolism might be an appealing option for the treatment of various blood cancers. Interestingly, MTX is routinely used only in the treatment of ALL and central nervous system (CNS) lymphoma, even though all blood cancer cell lines demonstrate high sensitivity. As further discussed below, limited application of MTX is linked to resistance mechanisms that cancer cells acquire to minimise the effectiveness of antifolates [8, 9]. For instance, AML blasts have a decreased ability to retain substantial MTX levels intracellularly [10]. Furthermore, antifolates, which broadly inhibit folate-mediate reactions, can manifest a series of side effects in normal proliferating tissue, such as damage to the gastrointestinal epithelium and impaired haematopoiesis [11, 12]. Resistance to antifolates and adverse side effects highlight the growing need for novel strategies to target 1C metabolism.

### Folate cycle and compartmentalisation

**Fig. 2:**
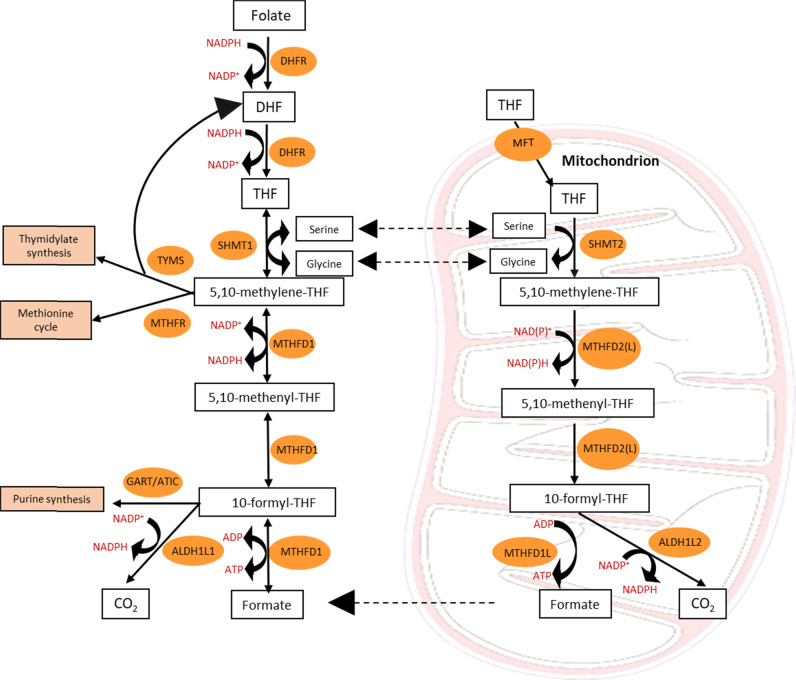
An overview of folate-mediated 1C metabolism and its compartmentalisation. From yeast to mammalian cells 1C units tend to flow clockwise allowing for a complete oxidative/reductive cycle where serine gets oxidised in the mitochondrial compartment and formate gets reduced in the cytosol. Through this intercompartmental cycle folate metabolism supports anabolic reactions such as purine and thymidylate synthesis as well as the methionine cycle. DHF, dihydrofolate; THF, tetrahydrofolate; DHFR, dihydrofolate reductase; MFT, mitochondrial folate transporter; SHTMT1/2, serine hydroxymethyl transferase, cytosolic (1)/ mitochondrial (2); MTHFD1, methylenetetrahydrofolate dehydrogenase 1; MTHFD2(L), methylenetetrahydrofolate dehydrogenase 2 (2-like); MTHFD1L, monofunctional tetrahydrofolate synthase; TYMS, thymidylate synthetase; MTHFR, methylenetetrahydrofolate reductase; GART, phosphoribosylglycinamide formyltransferase; ATIC, 5-aminoimidazole-4-carboxamide ribonucleotide formyltransferase/IMP cyclohydrolase; ALDH1L1/2, 10-formyltetrahydrofolate dehydrogenase cytosolic (1)/mitochondrial (2).

Folate metabolism occurs in two parallel and complementary pathways, localised in the cytosol and mitochondria (Fig. 2). New 1C units are donated from amino acids such as serine (the primary source of 1C units), glycine (through the glycine cleavage system) and the choline degradation products dimethylglycine and methylglycine (sarcosine), while folate molecules function as 1C unit carriers. In brief, the first step of the reaction is catalysed by serine hydroxymethyl transferase, SHMT1 (cytoplasmic) and SHMT2 (mitochondrial), where serine gets converted to glycine and its 1C unit is donated to tetrahydrofolate (THF) to give rise to 5,10-methylene-THF. In the cytosol, 5,10-methylene-THF contributes to thymidylate synthesis and the methionine cycle. Interestingly, SHMT2 was found to contribute to mitochondrial translation by providing methyl donors for the taurinomethyluridine synthesis [13]. 5,10-methylene-THF is then converted to the most oxidised form of folate carbon, 10-formyl-THF. In the cytosol, this conversion is carried out by the trifunctional enzyme methylene-THF dehydrogenase 1 (MTHFD1) with a 5,10-methylene-THF dehydrogenase, 5,10-methenyl-THF cyclohydrolase and 10-formyl-THF synthase activity. Whereas in the mitochondria, it is the bifunctional methylene-THF dehydrogenase 2 (MTHFD2) or 2 like (MTHFD2L) with dehydrogenase and cyclohydrolase activity that catalyses the conversion. Nilsson et al. [14] demonstrated that normal and cancer cells express both enzymes, even though *MTHFD2* exhibits a higher baseline expression. Surprisingly, *MTHFD2L* exhibited a greater increase in cancer tissue versus normal tissue in AML compared to *MTHFD2*,. In general, MTHFD2L was unable to compensate for the loss of MTHFD2 and it did not show any association with proliferation or response to growth factors. Cytosolic 10-formyl-THF is essential for de novo purine synthesis. More specifically, for every newly synthesised DNA or RNA base, one molecule of 10-formyl-THF is required [2, 15]. Mitochondrial 10-formyl-THF is used to initiate mitochondrial translation by producing formyl-methionine tRNA. Additionally, as 10-formyl-THF cannot cross the mitochondrial membrane, it first gets hydrolysed to formate by the MTHFD1-like enzyme. Lastly, 10-formyl-THF can be fully oxidised to CO_2,_ a reaction catalysed by 10-formyltetrahydrofolate dehydrogenase 1 (ALDH1L1) or 2 (ALDH1L2) in the cytosol or mitochondria respectively, leading to its terminal elimination.

The presence of these two parallel pathways allows for a complete oxidative/reductive cycle, where serine gets catabolized in the mitochondria and then synthesised in the cytosol. Interestingly, studies have revealed that this subcellular compartmentalisation is conserved from yeast to mammalian cells [16]. Ducker et al. [17] using a serine tracer that was developed to distinguish the mitochondrial-derived serine contribution versus the cytosolic [18, 19], demonstrated that most cultured cell lines generate 1C units exclusively in the mitochondria and that mitochondrial-released formate directly contributes to nucleotide and serine synthesis. Interestingly, the same and consecutive studies have shown that genetic inhibition of mitochondrial 1C enzymes induces a rewiring of the flux, where the cytosolic pathway switches from serine synthesis to serine catabolism [17, 20]. Cells end up becoming glycine auxotrophs, depending on extracellular glycine, as the cytosolic pathway cannot compensate for the loss of mitochondrial glycine production [21]. Labuschagne et al. [22] showed that in the absence of serine, exogenous glycine is unable to support nucleotide synthesis as cells start using glycine for serine synthesis.

A number of studies have tried to shed light on the compartmentalisation and directionality of folate metabolism. Conversion of 5,10-methylene-THF to 5,10-methenyl-THF by MTHFD2 is accompanied by mitochondrial NAD(P)H production. Whereas the reverse reaction catalysed by MTHFD1 in the cytosol proceeds in a NADPH-consuming direction [17, 23, 24]. The difference in electrochemical potential between NADH and NADPH explains the directionality of folate metabolism, where mitochondrial formate gets shuttled to the cytosol. With regards to why there should be a mitochondrial compartment in the first place, Zheng et al. [25] conclude that the existence of the mitochondrial compartment provides the necessary flexibility to balance cellular demands for glycine and 1C units. Whereas, a single cytosolic compartment would probably overwhelm the cell, as it would require multiple complex regulatory mechanisms (similar to *E. coli* where there is a single compartment). Additionally, Ducker and Rabinowitz [2] suggest that most of serine catabolism is localised in the mitochondria to separate 1C metabolism from glycolysis that happens in the cytosol. They argue that NADH production from the conversion of 5,10-methylene-THF to 5,10-methenyl-THF could deplete the cytosolic NAD^+^ pool needed for glycolysis. Overall, it seems that nature and evolution have enabled this compartmentalisation to maximise efficiency.

### Formate overflow and its implications

Several studies have reported that formate production through serine catabolism exceeds the 1C demands for nucleotide synthesis. This excess of formate, also known as formate overflow, depends on the expression of mitochondrial folate enzymes and the oxidative phosphorylation status of the cells [17, 20, 26, 27]. Meiser et al. [27] demonstrated that tumours with an active and robust oxidative metabolism are formate overflow positive, describing this formate overflow as a hallmark of oxidative cancers. Additionally, the authors revealed that cancer tissues exhibit significantly higher serine catabolism to formate, compared to normal adjacent tissue and other non-tumour-bearing organs. Plasma formate levels were also found to be significantly higher in tumour bearing mice compared to wild-type mice.

There are various hypotheses for the selective advantage of formate overflow, but it is still difficult to make solid conclusions. When Meiser et al. [27] demonstrated that formate overflow is a hallmark of oxidative tumours they also showed that the excess formate released from cancer cells promoted invasion in vitro. Here it is tempting to hypothesise that increased mitochondrial 1C metabolism and consequently formate overflow, might be advantageous for cancer development as it promotes invasion. Furthermore, Oizel et al. [28] conclude that formate overflow induces a metabolic switch to a cellular state with high purine and pyrimidine nucleotide levels and an increased rate of glycolysis. The increase in purine synthesis links formate overflow with cell signalling. In the process of increasing purine synthesis, there is a dramatic reduction of intracellular 1-(5′-Phosphoribosyl)-5-aminoimidazole-4-carboxamide ribotide (AICAR), the phosphorylated variant of the AMP activated kinase (AMPK) activating drug acadesine. The decrease in AICAR then results in a reduction of AMPK activity [28]. Concomitantly, the increase in purine levels promotes an increase in the mammalian target of rapamycin (mTOR) kinase activity, the master regulator of cell growth [29]. Ultimately, what they hypothesise is that serine catabolism to formate is required to trigger this metabolic switch, which potentially serves as a selective advantage for the existence of the mitochondrial branch of folate metabolism.

### NADH/NADPH production and its implications

Undeniably, the major role of folate metabolism is its contribution to nucleotide synthesis. However, more and more evidence suggest that it is not just nucleotide contribution that might have important implications on a cellular level. Similar to the formate overflow, and its role in energy metabolism (as a metabolic switch) and invasion, there is increasing evidence that other by-products of the folate pathway have important roles in the cell.

NADH production by the mitochondrial MTHFD2 links 1C metabolism to oxidative phosphorylation. NADH is subsequently oxidised while its electrons are transferred to the electron transport chain (ETC) complexes. This, ultimately, contributes to energy production in the form of ATP. Remarkably, Ducker et al. [17] demonstrated that serine catabolism contributes to up to 7% of the NADH pool. In addition, formate production correlates with the respiration status of the cell, where ETC impairment upon treatment with ETC inhibitors (i.e. rotenone, metformin) or mitochondrial DNA depletion, causes a reduction of formate release from the cells [20, 21, 26]. Interestingly, Bao et al. [26] showed that respiration chain dysfunction induces an adaptive response to increase serine synthesis (1C donor) to feed the mitochondrial 1C pathway. Furthermore, Yang et al. [30] described that upon respiration impairment, serine catabolism becomes a major source of NADH, whereas the other NADH sources like glutamine, fatty acid oxidation and lactate are blocked. This persistence of serine catabolism contributes to an accumulation of NADH that can be toxic. Inhibition of serine catabolism in the context of impaired respiration can normalise NADH levels and facilitate cell growth. However, previous studies have reported a synergistic effect in growth inhibition when 1C metabolism (via serine deprivation) and ETC are impaired [31, 32]. What one can say with certainty is that there is a definitive link between cellular respiration and 1C metabolism and it is worth of further investigation.

The oxidation step of methylene-THF to 10-formyl-THF is coupled to production of NADPH. In addition, full oxidation of 10-formyl-THF to CO_2_ also contributes to NADPH [23, 24]. NADPH is primarily synthetised through the pentose phosphate pathway (PPP) and provides high-energy electrons for antioxidant defence and reductive biosynthesis such as fatty acid and proline synthesis. Interestingly, to date there are two connections of folate metabolism to NADPH. The first connection is the production of NADPH via 1C metabolism and the second is that NADPH production through the PPP supports the folate pathway via regulation of the dihydrofolate reductase (DHFR) activity [33]. In a cancer context, evidence suggests that upregulation of mitochondrial 1C metabolism contributes to cancer progression by supporting an antioxidant defence. Ye et al. [34] described that in *MYC* amplified cancer cell lines and neuroblastoma patient samples, *SHMT2* gets induced to produce NADPH (by initiating the degradation of serine to CO_2_) and to maintain redox balance under hypoxic conditions. Ducker et al. [17] also demonstrated that cell lines deficient in *MTHFD2* and *ALDH1L2* were sensitised to hydrogen peroxide, a form of oxidative stress. Piskounova et al. [35] showed that folate pathway inhibition by MTX, *ALDH1L2* knockdown or *MTHFD1* knockdown inhibited tumour metastasis, concluding that ALDH1L2 counteracts oxidative stress and promotes metastasis. On that note, it was recently shown that cybrid cells (cells produced by combining cytoplasm of nucleated cells with non-nucleated cells or cytoplasts) with a mutation in a gene encoding an integral component of Complex I subunit of the ETC display a reduction in serine catabolism and NADPH production that leads to oxidative stress, inflammation and ultimately, cell death [36]. While in HCT116 cells, both *ALDH1L1* and *ALDH1L2* expression is minimal, suggesting that the folate pathway does not contribute substantially to NADPH, *ALDH1L2* expression is found to be upregulated in several tumour types [37]. It would be interesting to gain a better insight into this upregulation that suggests that specific tumours would require NADPH for an antioxidant response, therefore making them more vulnerable to oxidative stress.

### Targeting 1C metabolism in blood cancers

Since the late 40 s when Sydney Farber administered a newly synthesised folate analogue (aminopterin) to children with ALL, antifolates targeting 1C metabolism have been used to treat a wide range of cancers including haematological malignancies (Table 1). MTX, the most frequently used antifolate, has been approved by the Food and Drug Administration for the prophylaxis of meningeal leukaemia and it is also used in maintenance therapy with other chemotherapeutic drugs in patients with ALL [38]. MTX treatment, followed by whole-brain radiation, is the mostly commonly used approach for patients diagnosed with a rare form of non-Hodgkin lymphoma, CNS lymphoma [39].

**Table 1:** Antifolates and their application in haematological cancers.

Drug Name	Enzymatic target	Clinical application	Reference
*Methotrexate*	DHFR, TYMS and GART/ATIC	prophylaxis of meningeal leukaemia maintenance therapy in ALL CNS non-Hodgkin lymphoma advanced non-Hodgkin lymphoma Burkitt’s lymphoma diffuse large B-cell lymphoma relapsed and refractory ALL advanced mycosis fungoides (type of cutaneous T-cell lymphoma)	Izbicka et al. [38] Ferreri et al. [39] Batchelor et al. [103] Schmiegelow et al. [91] Kloos et al. [80]
*Pralatrexate*	DHFR, TYMS and GART/ATIC	refractory T-cell lymphoma preclinical models of multiple myeloma	Sirotnak et al. [87] Kinahan et al. [88]
*Pemetrexed*	DHFR, TYMS and GART/ATIC	preclinical models of multiple myeloma	Ramirez et al. [89]
*SHIN1* *SHIN2*	SHMT1/2	T-ALL (preclinical) diffuse large B-cell lymphoma (preclinical)	García-Cañaveras et al. [97] Pikman et al. [98]

**Fig. 3:**
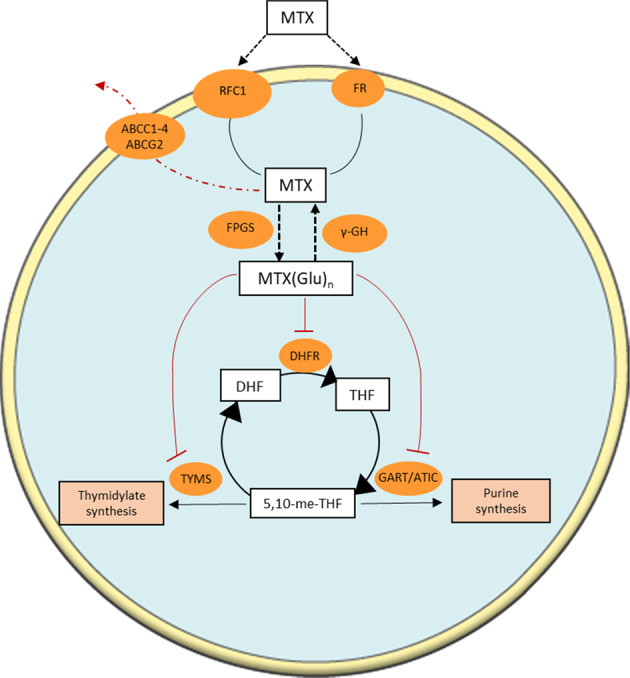
Mechanism of action of methotrexate. Methotrexate (MTX) enters the cell mainly through the reduced folate carrier (RFC1) and to a lesser extend through receptor-mediated endocytosis via a folate receptor (FR). Upon entry, MTX gets polyglutamated (MTX(Glu)_n_) by folylpolyglutamate synthase (FPGS). Polyglutamates of MTX are a superior antifolate agent compared to MTX, capable of highly potent irreversible inhibition of DHFR. Furthermore, MTX induces inhibition of other enzymes like TYMS and GART/ATIC, ultimately blocking de novo thymidylate and purine syntheses. γ-glutamyl hydrolase (γ-GH) (compartmentalised in lysosomes) removes glutamate residues from MTX, while ATP-binding cassette (ABC) transporters assist in the excretion of MTX from the cell.

MTX enters the cell via the reduced folate carrier (RFC1), encoded by *SLC19A1*, a protein found predominantly in the cell membranes of foetal and cancer cells (Fig. 3). Once in the cytosol, it gets polyglutamated by folylpolyglutamate synthetase. Polyglutamation is a time- and concentration-dependent process that ultimately enhances the action of MTX. Polyglutamates (up to five additional glutamates) of MTX are retained longer in the cell, thus the amount accumulated and the length of the chain of MTX polyglutamates are key determinants of antifolate-mediated cytotoxicity. MTX and its polyglutamates inhibit DHFR, that catalyses the conversion of dihydrofolate to THF. Additionally, polyglutamation alters the cellular effects of MTX, as studies have shown that MTX polyglu tamates are more potent inhibitors of other folate-dependent enzymes such as thymidylate synthase (TYMS) and 5-aminoimidazole-4-carboxyamide ribonucleotide formyltransferase, ultimately blocking both thymidylate and purine syntheses [38, 40–42]. MTX polyglutamates with chains longer than 3 glutamate residues cannot be exported out of the cell, therefore, they get deglutamated by the lysosomal γ-glutamyl hydrolase. The balance between polyglutamation and deglutamation generates a steady state level of intracellular MTX. Lastly, MTX gets exported from the cell by the ATP-binding cassette proteins (ABCC1-4 and ABCG2). ATP-binding cassette proteins play a unique role as they form a defence network against a number of chemotherapy agents and cellular toxins [43]. Furthermore, overexpression of these efflux transporters has been associated with antifolate resistance [44].

### Cellular effect of MTX treatment

**Fig. 4:**
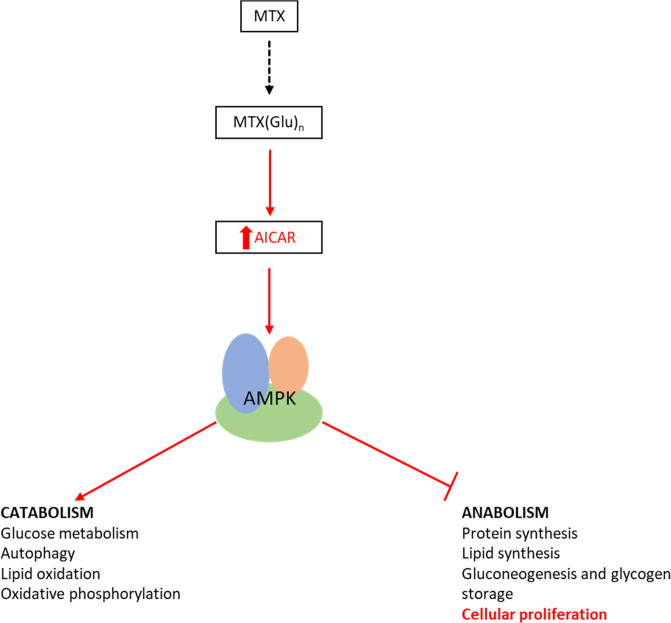
Metabolic changes caused by MTX treatment. MTX by inhibiting de novo purine synthesis causes significant accumulation of AICAR. AICAR is an activator of 5’ AMP-activated protein kinase (AMPK), a master regulator of energy homoeostasis. AMPK signalling promotes catabolic pathways such as glycolysis, autophagy, lipid oxidation and oxidative phosphorylation etc. to augment cellular bioenergetic capacity. Concurrently, AMPK inhibits anabolic processes such as protein and lipid synthesis and ultimately restrains cellular proliferation.

A number of studies have aimed to further describe the mechanism of action of MTX. MTX treatment causes accumulation of AICAR leading to activation of AMPK in breast and prostate cancer cell lines [45]. Induction of AMPK through MTX was found to promote glucose uptake and lipid oxidation, as well as inhibiting protein synthesis, linking the anti-proliferative effects of MTX to AMPK activation [45–47] (Fig. 4). Interestingly, Savino et al. [48] demonstrated that leukaemic cells that migrate to CNS upregulate fatty acid synthetic pathways. It could be hypothesised that MTX-induced fatty acid oxidation can have an antiproliferative effect in these cells that are depending on fatty acids.

Sengupta et al. [49], while investigating the effect of AICAR in ALL cell lines, found that AICAR-mediated AMPK activation inhibited cell proliferation, induced cell cycle arrest and apoptosis. In addition, cells treated with AICAR exhibited activation of the phosphatidylinositol 3-kinase/protein kinase B (PI3K/Akt) signalling pathway and inhibition of mTOR. Beckers et al. [50] suggested that MTX enhances the antianabolic and antiproliferative effects of cell permeable AICAR treatment, where MTX and AICAR treatment were found to have a synergistic effect. Similarly, Kuznetsov et al. [51] demonstrated that AICAR potentiates MTX-induced ROS accumulation and consequently ER stress. They hypothesise that AICAR in combination with MTX treatment, and in conjugation with the upregulation of PI3K/Akt pathway that inhibits AMPK, leads to prolonged cellular ER stress that ultimately results in cell death. While PI3K/Akt pathway normally exhibits a pro-oncogenic role, in this model it seems to play a pro-apoptotic role. Furthermore, mTOR inhibitors have been found to downregulate DHFR expression and increase sensitivity to MTX both in vitro and in vivo. These findings also led to a pilot clinical trial that aimed to investigate such combination treatment in adults with relapsed ALL [52]. As one of the main mechanisms of MTX resistance is upregulation of DHFR expression, the fact that mTOR inhibitors might have an effect on this enzyme is something that needs to be further investigated.

Papadopoli et al. [53] demonstrated a similar effect when MTX was combined with biguanides (metformin, phenformin; display AMPK-dependent anti-tumorigenic effects), stimulating discussion on how biguanides can be used to improve chemotherapeutic response to antifolates. Indeed, there has been increasing interest in repurposing biguanides for cancer treatment. Interestingly, at clinically relevant concentrations, metformin was found to induce folate cofactors to become trapped as 5-formimino-THF, a cofactor that cells seem to be unable to use for 1C units, ultimately blocking DNA and RNA synthesis. This antifolate behaviour of metformin can potentially present a major mechanism of its anticancer effect, linked to an AMPK-dependent anti-neoplastic response [54]. The fact that metformin behaves as an antifolate and activates AMPK might explain why it synergises with MTX. In addition to its antitumour activity, metformin was found to ameliorate MTX-induced hepatorenal toxicity in an in vivo model. Kanarek et al. [55] suggested that the histidine degradation pathway also influences the sensitivity to MTX in leukaemia xenografts, suggesting that histidine dietary intervention could potentially be exploited to improve efficacy.

### Clinical application of MTX

It is worth mentioning that usually patients with ALL and lymphoma receive intravenously delivered MTX doses of 500 mg/m^2^ or higher, known as high-dose MTX. Such dosages are routinely used to treat CNS ALL, where conventional doses of MTX fail to protect against CNS relapse. Doses of up to 33.6 g are being utilised to provide significant MTX exposure in sites like CNS [56–59]. High-dose MTX treatment can have adverse effects in patients, leading to significant morbidity and even mortality [60]. Toxicity is primarily caused by delayed MTX excretion and persistent high plasma MTX levels as a result of MTX-induced renal dysfunction [61]. Gastrointestinal mucosal injury, bone marrow suppression, renal or liver impairment are some of the adverse effects caused by high-dose MTX [11, 12]. Nilsson et al. [62] showed that DHFR and TYMS are expressed in many proliferating tissues, in which the adverse effects of MTX are observed. In agreement with the DHFR and TYMS expression pattern, MTX has been shown to accumulate in bone marrow cells and intestinal mucosa both in vitro and in vivo. Even though malignant cell lines have shown a markedly greater capacity to convert MTX to polyglutamates, emergence of MTX polyglutamates is dose and time dependent, thus making highly replicating cells, with high demands of purine and thymidylate synthesis, susceptible to MTX-induced toxicity [63–66]. Furthermore, Barredo and Moran [67] described that FPGS activity is linked to proliferation rate and differentiation stage. In other words, highly proliferating tissues and immature cells exhibit an increased activity of FPGS. These findings can give some clues about the susceptibility of these tissues to antifolates; however, it would be interesting to further assess differences of FPGS, FPGH activity, expression of MTX influx and efflux transporters between malignant cells and other highly proliferating cells.

### Approaches to minimise MTX toxicity

Combination treatments of MTX with leucovorin (folinic acid), or lower doses of MTX, are strategies that have been developed to minimise adverse effects. Nevertheless, low-dose MTX has been reported to cause pneumonitis and leukoencephalopathy in patients with rheumatoid arthritis, where MTX is a standardised treatment [12, 68]. Leucovorin is a structural analogue of MTX, thus it can compete for membrane transport, polyglutamation and binding to DHFR. However, as it does not require reduction by DHFR, it can restore reduced folate pool even in the presence of MTX [69, 70]. MTX polyglutamates have the tendency to persist in malignant and normal cells even after the removal of extracellular MTX, making the addition of leucovorin essential for the rescue of normal tissues [71]. Nonetheless, in vitro and in vivo studies have demonstrated that high doses of leucovorin associate to higher risk of relapse possibly by rescuing malignant cells from the effect of high-dose MTX [70, 72, 73]. Skärby et al. [58] using the Nordic Society of Paediatric Haematology and Oncology database showed that high doses of leucovorin during a high-dose MTX regime correlated with an increased risk of relapse in ALL patients. Surprisingly, these patients demonstrated high plasma MTX levels and longer elimination time, which normally correlate with a better prognosis.

Administration of glucarpidase followed by leucovorin treatment is another strategy that has been exploited in patients who do not demonstrate the expected MTX clearance. Glucarpidase cleaves MTX into two nontoxic metabolites, 2,4-diamino-N-10-methylpteroic acid and glutamate, providing a method to rapidly remove MTX in patients with renal dysfunction. It rapidly decreases plasma MTX, but it does not have any effect in intracellular MTX concentration, thus patients still need to receive leucovorin. Vigorous hydration and urinary alkalinization prior to MTX infusions are additional care measures that are routinely used to minimise toxicity [60]. Furthermore, a concomitant treatment regime of MTX with asparaginase has been developed to treat patients with relapsed and refractory ALL. In addition, Buaboonnam et al. [74] demonstrated that the combination of the two agents had efficacy in 6 out of 15 children with refractory or relapsed AML after salvage chemotherapy. Asparaginase is thought to act as a ‘rescue’ agent and permit administration of normally toxic doses of MTX [75, 76]. Early in vitro studies stressed out the importance of the sequence of the administration of the two agents, as asparaginase was shown to have antagonistic effect to MTX when it is administrated prior to the antifolate [75, 77, 78]. However, recent studies have suggested that schedule-related antagonism might not be as important in vivo [79, 80]. Asparaginase does not seem to contribute to additional MTX toxicity, but it also does not ameliorate any of the adverse effects of high-dose MTX [80].

### Antifolate resistance

Another issue that arises with MTX treatment is drug resistance. Mutations in *SLC19A1*, amplification or mutation of *DHFR*, and loss of polyglutamation have been shown to contribute to the loss of efficacy [8, 9, 42]. Apart from acquired resistance to MTX, an interesting aspect is the intrinsic resistance. Even though AML cell lines show high sensitivity to MTX (Fig. 1) blast cells from AML patients appear to have a reduced ability to form long-chain polyglutamates compared to cells from ALL patients, making them unable to retain substantial amounts of MTX [10]. This inability to form long-chain polyglutamates has been linked to increased expression of ABC efflux transporters, low activity of FPGS and high activity of γ-GH, responsible for the degradation of the glutamate side chain [81–83] (Fig. 3). Interestingly, a number of studies have described that MTX polyglutamation in samples from acute monocytic leukaemia patients (AML-M5) and acute megakaryocytic leukaemia (AML-M7) patients is comparable to paediatric B-ALL patient samples [84, 85]. Rots et al. [86] observed poor MTX polyglutamation in AML-M5 samples, but an increases sensitivity to MTX compared to AML-nonM5. In support to this, a closer look to the AML cell lines in the Genomics of Drug Sensitivity in Cancer database (https://www.cancerrxgene.org/) (Fig. 1) demonstrates that AML-M5 cell lines (e.g. THP1, MOLM13, MONO-Mac-6) show pronounced sensitivity to MTX, contributing directly to the overall high sensitivity of AML cell lines to MTX. Further investigation with regards to MTX polyglutamation in AML-M5 is required to evaluate the possible use of MTX as a treatment. However, intrinsic resistance is a noteworthy issue, as it points to the critical need of new ways to target 1C metabolism that could potentially overcome the need for intracellular polyglutamation.

Pralatrexate (PDX), a MTX analogue, was designed to have improved cellular transport via RFC-1 and enhanced formation of polyglutamates, resulting in greater intracellular retention [87]. In 2009, PDX was approved for the treatment of relapsed or refractory T-cell lymphoma and in a recent study it was shown to have anti-tumour activity in preclinical models of multiple myeloma (MM) [88]. In addition, pemetrexed, another anti-folate that shows high affinity for both DHFR and TYMS, was described to cause cell cycle blockage and consequently apoptosis in MM cells in vitro and also in animal models in vivo [89]. However, pemetrexed and pralatrexate target predominantly DHFR implying that they can have similar limitations regarding toxicity in normal tissues and drug resistance as MTX.

### Additional purine and pyrimidine synthesis inhibitors

Inhibitors that directly target purine or pyrimidine synthesis has also been applied in haematological malignancies. 6-mercaptopurine (6-MP), is a thio-substituted purine analogue that inhibits de novo purine synthesis. Burchenal et al. [90] demonstrated that 6-MP induced temporary remission in childhood leukaemia. Together with MTX, 6-MP is universally used as maintenance therapy in childhood ALL [91]. This purine analogue is converted into 6-thioguanine nucleotide and 6-methylmercaptopurine (6-MMPN), whose concentration impacts drug response and toxicity. However, during 6-MP/MTX maintenance therapy, increased levels of 6-MMPN correlate with hepatotoxicity, a frequent side effect of the therapy [92]. In addition, inhibitors of de novo pyrimidine synthesis have shown promising results in blood cancers. More specifically, inhibitors of dihydrooratate dehydrogenase (DHODH), an enzyme located in the inner membrane of the mitochondria that catalyses the fourth step of de novo pyrimidine synthesis, have emerged as therapeutic targets in AML and MM. Leflunomide, one of the DHODH inhibitors developed, was found to induce cell cycle arrest and apoptosis through inhibition of c-Myc [93, 94]. Recently, a novel DHODH inhibitor was shown to induce differentiation in different models of AML in vitro and to prolong survival in AML animal models [95].

### Emerging strategies to target 1C metabolism in blood cancers

**Fig. 5:**
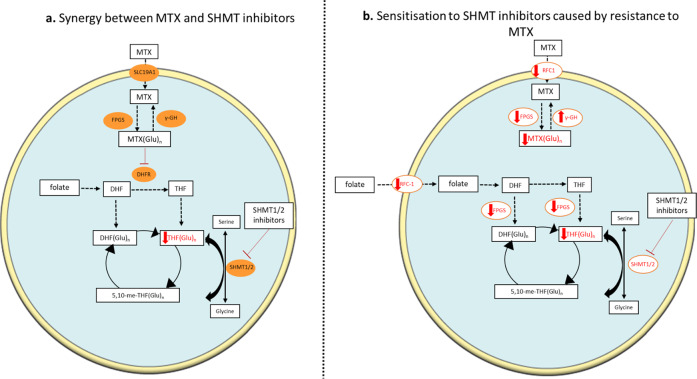
Hypothesised mechanisms of action of SHMT inhibitors. **a** Proposed mechanism of synergy of MTX with SHMT inhibitors. Inhibition of DHFR by MTX decreases intracellular THF, thus, it sensitises cells to SHMT inhibitors. **b** Proposed mechanism of increased sensitivity to SHMT inhibitors due to MTX resistance. Decreases transport mediated by RFC1, decreased polyglutamation due to decreased activity of FPGS or increased activity of γ-GH are mechanisms that contribute to MTX resistance. Decreased MTX(Glu)n correlates with decreased THF(Glu)n as folate has a similar influx mechanism and polyglutamation pattern. As THF is a substrate for the SHMT reaction, depletion of this substrate leads to increased sensitivity of the cells to the SHMT inhibitors.

A number of recent studies have tried to reveal novel strategies to target 1C metabolism. Nilsson et al. [62] demonstrated that among 1454 metabolic enzymes examined, MTHFD2 is the most consistently overexpressed in tumours, while SHMT2 is the fifth. Vazquez et al. [96] proposed that expression of mitochondrial 1C metabolism enzymes determine the response to MTX. On that note, García-Cañaveras et al. [97] showed that a dual inhibitor of SHMT1/2, SHIN2, synergises with MTX both in vitro and in vivo. More specifically, as a single agent SHIN2 inhibited the proliferation of T-ALL cell lines and increased survival in NOTCH1-driven mouse T-ALL models. Furthermore, it sensitised MTX-resistant human T-ALL cells. In support of this study, Pikman et al. [98] showed that T-ALL cell lines that had developed resistance to MTX, remained sensitive to dual inhibition of SHMT1/2. The authors also demonstrated that loss of both SHMT1 and SHMT2 activity was required for the anti-proliferative effect that was observed, whereas repression of each enzyme alone was not enough to elicit such an effect. The findings reported reinforce the idea that there is a redundancy between SHMT1 and SHMT2 and one can compensate for the loss of the other. Thus, to block production of 1C units from serine, simultaneous inhibition of both enzymes is necessary. It can be hypothesised that the synergistic effect of MTX with SHMT inhibitors might be a result of the depletion of THF (SHMT substrate) by MTX, therefore sensitising cells to SHMT inhibition (Fig. 5a). In MTX resistant cells, decrease of MTX polyglutamates due to decreased RFC1 or FPGS activity or increased γ-GH would result in decrease of THF-polyglutamates, as folate has a similar influx mechanism and polyglutamation pattern. Therefore, a decrease in THF-polyglutamates due to the MTX resistance would deplete the substrate of the SHMT reaction and make cells sensitive to SHMT inhibitors (Fig. 5b).

In the context of lymphoma, diffuse large B-cell lymphoma was shown to have a defective glycine import, making it sensitive to pharmacological SHMT inhibition [99]. This effect is explained by the fact that inhibition of mitochondrial metabolism makes cells glycine auxotrophs, depending on extracellular glycine uptake. In such circumstances, inhibition of the mitochondrial compartment would be enough to sensitise cells, as the cytosolic compartment cannot compensate for the lack of mitochondrial glycine production, as we previously mentioned. Lometrexol, an antifolate that targets GART, was found to have high affinity for SHMT2 [100]. Selective SHMT2 inhibitors could be useful for the treatment of malignancies like diffuse large B-cell lymphoma where glycine uptake is impaired and cancer cells are solemnly depending on mitochondrial 1C metabolism for glycine production. Lastly, it was reported that loss of MTHFD2 impaired growth and induced differentiation in AML cell lines and primary AML blasts, while it decreased leukaemia burden in human xenograft and MLL-AF9 mouse leukaemia models. In the same study authors described that AML cell lines characterised by a FMS-like tyrosine kinase 3 internal tandem duplication (*FLT3-ITD*) mutation are more sensitive to loss of MTHFD2 [101]. In an attempt to further characterise the metabolic changes in *FLT3-ITD* driven AML, Bjelosevic et al. [102] demonstrated that *FLT3-ITD* modulates serine synthesis and uptake as well as enzymes involved in 1 C metabolism.

The cancer cells response to SHMT1/2 inhibitors exhibits a metabolic pattern with many similarities to what observed for MTX. By depriving cells from 1C units and glycine, SHMT inhibitors block purine and thymidylate synthesis. SHMT inhibitors also promote an increase in AICAR levels and activation of AMPK [28], as observed for MTX. This evidence suggests that SHMT inhibitors have a mechanism of action similar to antifolates. Yet, by acting on a different enzyme and being independent of folate transporters, SHMT inhibitors may help to overcome resistance to MTX.

## Conclusion

Folate-mediated 1C metabolism comprises of a series of metabolic reaction that contribute to biosynthetic processes. As highly proliferating cells, including malignant cells, show dependence on folate metabolism to fulfil their biosynthetic needs, folate metabolism is increasingly gaining interest in the research community. Studies of the recent years have tried to shed light on the biological importance of the compartmentalisation of the pathway, formate overflow, as well as NADH and NADPH by-products. Its implications in cancer have given rise to modern cancer therapy. Antifolates, especially MTX, are being routinely used to treat various neoplastic diseases and they have found a significant application in many haematological malignancies. However, as we described above, side effects and resistance to antifolates are problems that are yet to be resolved, highlighting the need of new approaches to target this pathway.

Targeting enzymes associated with serine 1C metabolism might overcome the drug resistance that has been described for traditional antifolates. What makes them attractive is that they do not require any folate transporters or intracellular polyglutamation. Since 2017, when Ducker et al. [99] described the first pharmacological inhibitor of SHMT2/1, these inhibitors have shown promising results, especially in T-ALL leukaemia, as single agents but also in combination with MTX [97, 98]. These encouraging results could have implications in other blood cancers like AML and MM where effective treatment options are limited; however, this still requires further investigation. In addition, Oizel et al. [28] suggested that inhibition of SHMT2/1 causes accumulation of AICAR and activation of AMPK, as seen with MTX. Therefore, further understanding of the mechanism of action of these inhibitors would potentially help to explore new therapeutic approaches.
